# Polyamide 66 microspheres metallised with *in situ *synthesised gold nanoparticles for a catalytic application

**DOI:** 10.1186/1556-276X-7-182

**Published:** 2012-03-08

**Authors:** Nicolas Cheval, Nabil Gindy, Clifford Flowkes, Amir Fahmi

**Affiliations:** 1 Department of Materials, Mechanics and Structures, Faculty of Engineering, University of Nottingham, Nottingham, NG7 2RD, UK; 2 Faculty Technology and Bionics, Rhein-Waal University of Applied Sciences, Emmerich, D-46446, Germany

**Keywords:** polyamide 66, nanoparticle, catalyst, microsphere, gold, spherulites

## Abstract

A simple concept is proposed to metallise polyamide 66 (PA66) spherulite structures with *in situ *synthesised gold nanoparticles (Au NPs) using a wet chemical method. This cost-effective approach, applied to produce a PA66/Au NP hybrid material, offers the advantages of controlling the nanoparticle size, the size distribution and the organic-inorganic interactions. These are the key factors that have to be controlled to construct consistent Au nanostructures which are essential for producing the catalytic activities of interest. The hybrid materials obtained are characterised by means of scanning electron microscopy, transmission electron microscopy, attenuated total reflection-Fourier transform infrared spectrometry and X-ray diffraction spectrometry. The results show that PA66 microspheres obtained via the crystallisation process are coated with Au NPs of 13 nm in size. It was found that controlling the metal coordination is the key parameter to template the Au NPs on the spherulite surfaces. The preparation processes and the key factors leading to the formation of PA66 spherulites coated with Au NPs are discussed. Moreover, the efficiency of the coated spherulites as a potential catalyst is proved by demonstrating the reduction of methylene blue via UV-visible spectrometry.

## Introduction

Immobilising noble metal nanoparticles onto organic microsphere surface has been receiving a considerable amount of attention in chemical fields due to their interesting catalytic properties and useful practical applications [1-3]. Additionally, organic microspheres offer the advantage to be easily recycled by conventional filtration or centrifugation techniques [4-6]. Furthermore, it has been found that this spherical structure is an excellent support for promoting the intriguing catalytic properties of noble metal nanoparticles [7-9].

Over the last few years, various organic supports have been utilised to stabilise metallic nanoparticles [7-9]. For instance, Dokoutchaev et al. reported the fabrication of polystyrene (PS) microspheres coated with Au, Pt and Pd nanoparticles [10]. Whereas, Jeon et al. synthesised hierarchically structured microspheres composed of a PS-*b*-PEO diblock copolymer and gold nanoparticles [11]. These hybrid systems possess weak mechanical properties and low catalytic activities and are often complex to produce [12].

**Figure 1:**
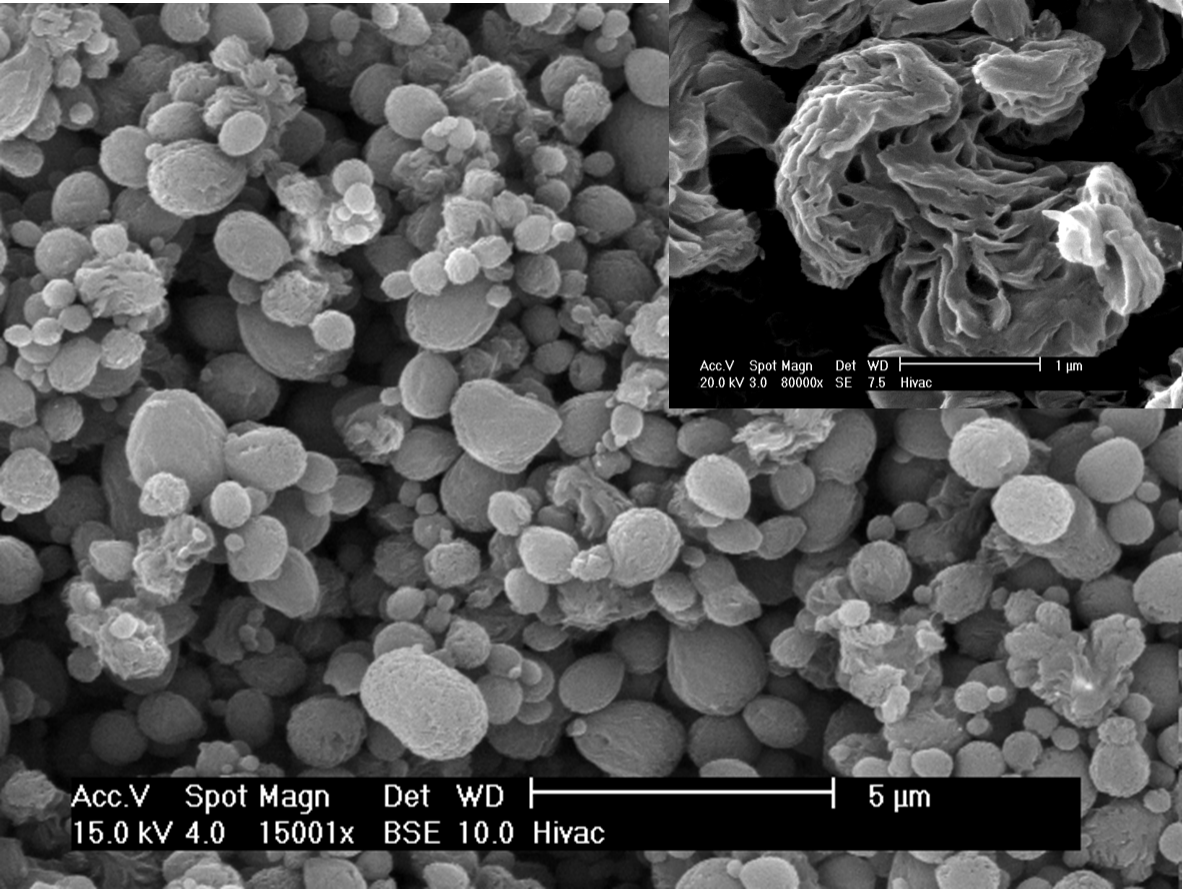
**SEM micrograph**. Neat PA66 spherulite obtained via crystallisation from solution.

Among all the polymers used as a support, a little attention has been devoted to polyamide 66 (PA66). This polymer, classified in the engineering semi-crystalline thermoplastic family, possesses excellent thermal, mechanical and chemical resistance properties [13]. Additionally, this type of polymer can easily form spherical nanostructures called spherulites via a crystallisation process from solution (Figure 1) [14]. Compared to PS microspheres which need to be functionalised by an amine or carboxylic acid group to coordinate the noble metal nanoparticles, PA66 has its polymer chain functional amide group to host a metal complex [8,12]. These well-defined PA66 functionalized spherulites could support noble metal nanoparticles onto their surfaces in order to collect their intriguing physical properties.

Controlling the nanoparticle diameter is found to be the key factor for harnessing their catalytic properties. Indeed, gold and palladium in bulk state are chemically inert but become chemically active for many reactions at a nanoscale level. It has been found that decreasing the particle size to nanoscale decreases their redox potential to a negative value [15-19]. Due to their high specific surface area and their low redox potential, stabilised noble metal nanoparticles are usually used in the chemical field as an effective catalyst [5,12].

Herein, we propose a simple concept to coat PA66 spherulites with gold nanoparticles (Au NPs) prepared *in situ *via a wet chemical approach. This cost-effective method offers the advantages of controlling the particle size, the size distribution and also the organic-inorganic interactions [20].

PA66/Au NP hybrid materials obtained were characterised by scanning electron microscopy (SEM), transmission electron microscopy (TEM), attenuated total reflection-Fourier transform infrared spectrometry (ATR-FTIR), energy-dispersive X-ray (EDX) analysis and X-ray diffraction (XRD) spectrometry. The catalytic property of PA66/Au NP microspheres was tested by investigating the reduction of methylene blue to leuco methylene blue in water medium using UV-visible (UV-Vis) spectrometry.

## Experimental details

### Materials

Commercial PA66 (Mw = 18,000 mg/ml) in pellet forms was supplied by Celanese Chemicals UK Ltd. (Stallingborough, UK). Gold chloride (HAuCl_4_), formic acid (HCOOH) and sodium borohydride (NaBH_4_) were purchased from Sigma-Aldrich Company Ltd. (Dorset, England, UK) and used as received.

### PA66 spherulite preparation

PA66 in a pellet form was dissolved in 98% formic acid. Distilled water was added slowly into the PA66 solution to crystallise the polymer in order to form spherulites (Figure 1). The powder recovered was dried for 3 h at 110°C. The PA66 average spherulite diameter size determined by dynamic light scattering (DLS) was estimated at 179.4 nm [see Additional file 1].

### Metallisation of PA66 spherulites

The PA66 powder recovered from the spherulite preparation process was mixed with the gold precursor (HAuCl_4_) solution in water. The mixture was stirred for 24 h to ensure the coordination between the organic and inorganic materials. The PA66/HAuCl_4 _system was then centrifuged and rinsed a few times to remove the excess of gold chloride. The system was re-dispersed in aqueous medium, and sodium borohydride was incorporated into the solution to reduce the gold precursor into Au NPs. Finally, the PA66/Au NP hybrid material powder was washed with distilled water to remove NaBH_4 _compound and subsequently dried at 110°C for 3 h to remove excess water.

### Measurements

Pristine and metallised PA66 spherulites were observed with a scanning electron microscope (model XL 30 ESEM FEG, Philips, Guildford, England, UK). PA66/Au NP microspheres were not coated to avoid the overlapping with the metal-conducting layer. EDX analysis was carried out on the sample at a working distance of 10 mm, using the INCA software (Oxford Instruments, Abingdon, UK). To assess the particle size and size distribution, the specimen was observed via a transmission electron microscope at 100 keV (Tecnai BioTWIN, FEI Ltd., Hillsboro, OR, USA). The TEM micrographs obtained were analysed with the program Gwyddion using the grain analysis function. UV-Vis spectra were recorded using a Varian Cary 50 photospectrometer (Varian Medical Systems UK Ltd., Crawley, UK) with the monochromator slit width of 10 nm. To determine the type of interaction between Au NPs and PA66, ATR-FTIR (Tensor 27, Bruker Optics, Rheinstetten, Germany) measurements were conducted at ambient temperature in the spectral range from 4,000 to 550 cm^-1 ^on the PA66/Au NP hybrid material powder recovered.

### Reduction of MB

A solution of methylene blue (MB) was prepared in aqueous medium at 1 mg/ml. One milligram of sodium borohydride (NaBH_4_) was added to 1 ml of MB solution. Optical absorption spectrum of the solution was measured every 4 min after the incorporation of NaBH_4 _by UV-Vis spectrometry. One milligram of the PA66/Au NP microsphere powder was added to the MB-NaBH_4 _solution. Then, the optical absorption spectrum of the solution was also recorded every 4 min to investigate the reduction of MB in the presence of PA66/Au microspheres.

## Results and discussion

**Figure 2:**
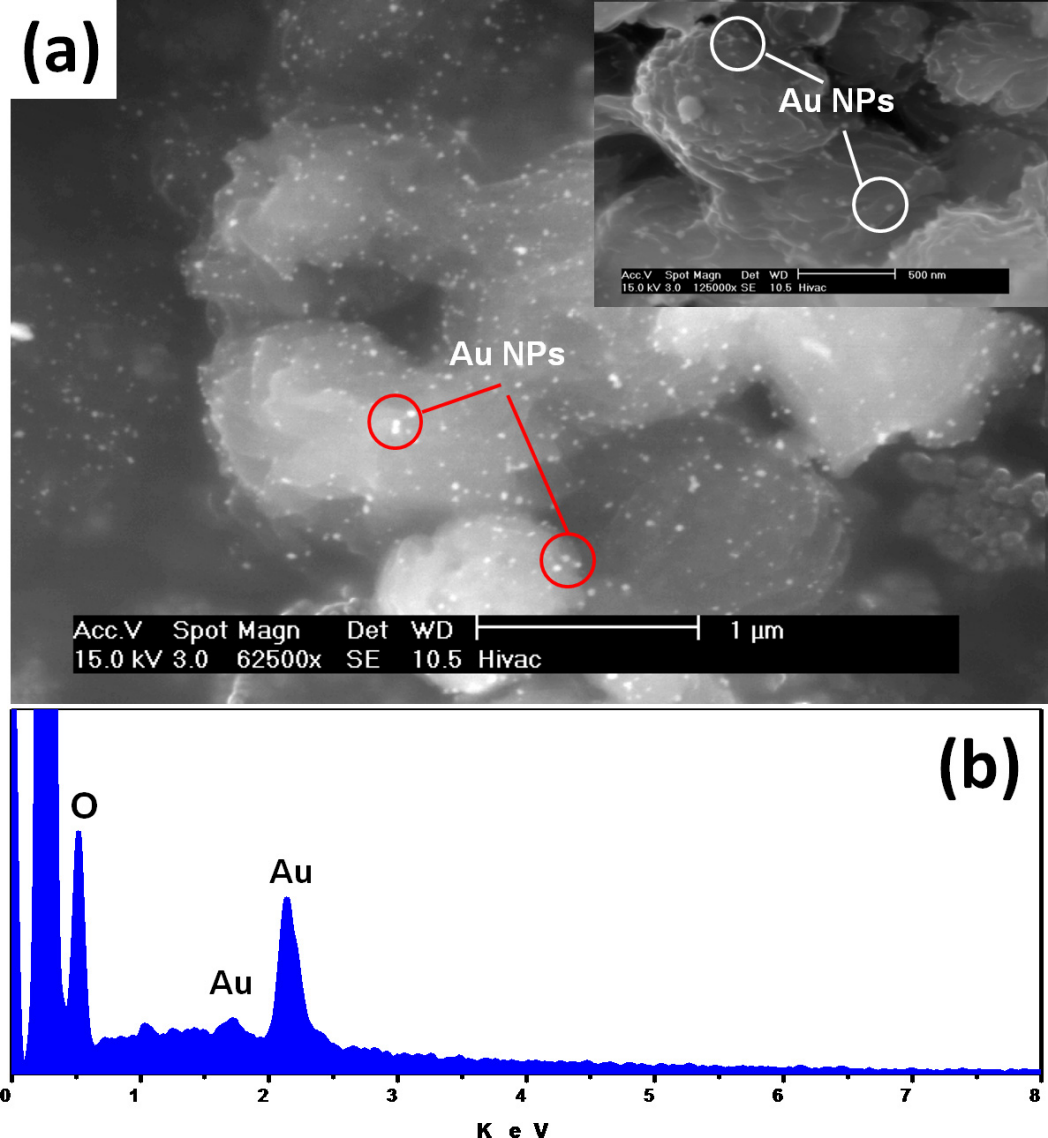
**SEM micrographs**. (a) PA66 metallised with Au NPs and (b) EDX analysis of PA66/Au NP microsphere surfaces.

PA66 spherulites coated with Au NPs were firstly observed via SEM (Figure 2). The sample was observed without coating to avoid the overlapping of the metal layer on the nanoparticles. SEM micrograph shows that PA66 microsphere surfaces are covered with spherical noble metal nanoparticles (Figure 2a). The presence of gold onto the PA66 spherulite surface was confirmed by conducting EDX analysis on the specimen surface (Figure 2b).

**Figure 3:**
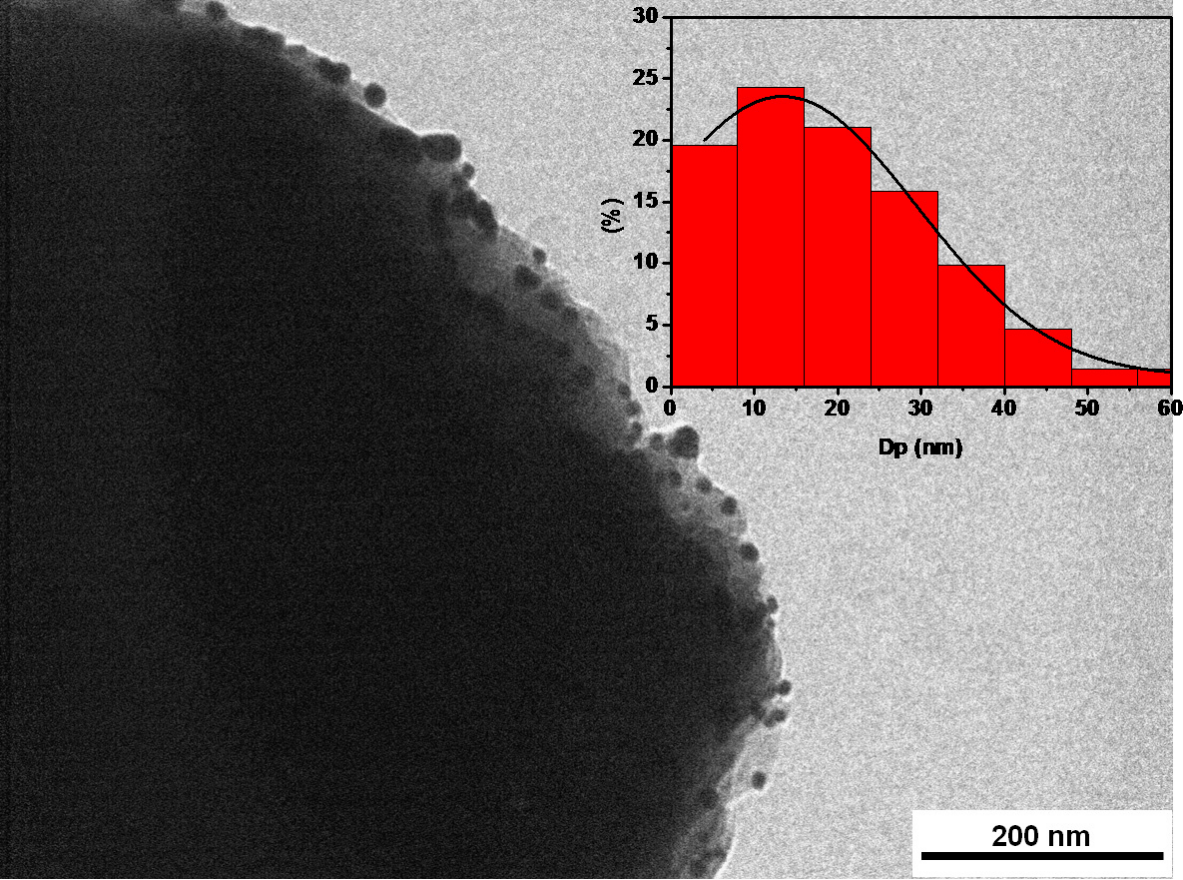
**TEM micrograph**. Microspheres of PA66/Au NP microspheres (the insert shows the size distribution of Au NPs).

As observed on the TEM micrograph, Au NPs synthesised *in situ *are located at the surface of the PA66 spherulites (Figure 3). The size and size distribution of Au NPs, which are the key parameters in the production of an effective catalytic nanoparticle, have been determined from TEM micrographs (insert in Figure 3). The average particle diameter of Au NPs was estimated at around 13 nm (insert in Figure 3). The SEM and TEM results demonstrate that the PA66 spherulite can be used as an effective support to stabilise Au NPs.

**Figure 4:**
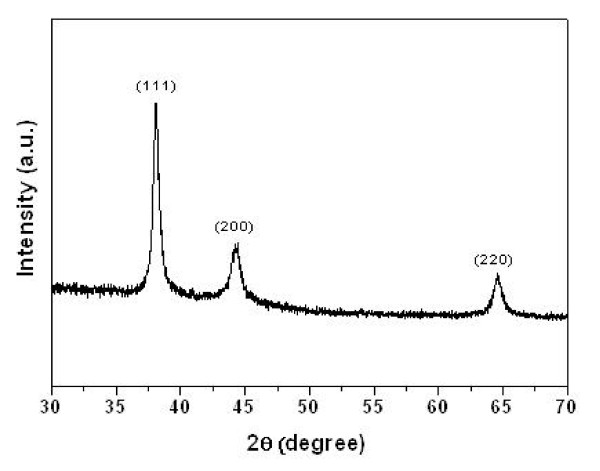
**XRD spectrum**. PA66 microspheres coated with Au NPs.

The crystalline structure of gold was accessed via XRD spectrometry. The XRD pattern of the PA66/Au NP hybrid material is displayed in Figure 4. The peaks, distinguished at 2*θ = *38.09°, 44.3° and 64.7°, are assigned to the (111), (222) and (220) lattice planes of gold in cubic structure [21]. The result demonstrates that the gold precursor is reduced to form crystallised Au NPs after adding NaBH_4 _into the solution.

**Figure 5:**
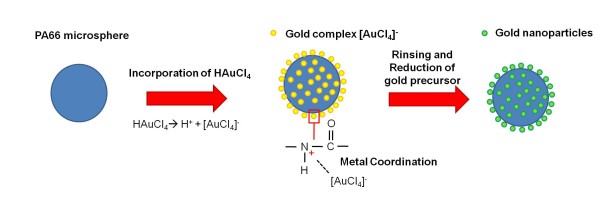
**Possible model of PA66 microspheres metallised with Au NPs prepared by a chemical approach**.

Based on the TEM and SEM results, a possible mechanism for the metallisation of PA66 spherulites with Au NPs is proposed in Figure 5. PA66 microspheres obtained by precipitation (Figure 1) were re-dispersed in water medium. Adding the gold precursor into the PA66 solution leads to the acidification of the medium since the gold precursor dissociates in water to form hydrogen ions (H^+^) and gold complex ([AuCl_4_]^-^). Thus, the amide group, protonated at the spherulite surface by the hydrogen ions into the solution, can interact with the gold complex charged negatively [22] (Figure 5). The metal coordination is the key factor to anchor Au NPs at the PA66 surfaces after the reduction process.

**Figure 6:**
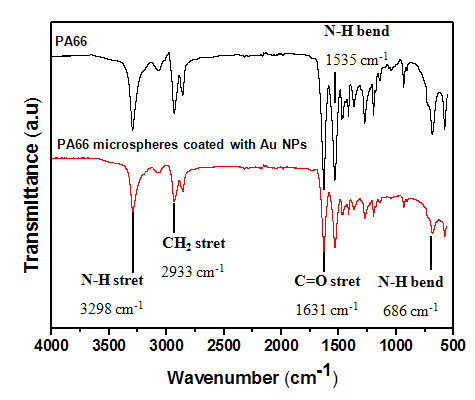
**ATR-FTIR spectrum**. PA66 and PA66 microspheres coated with Au NPs.

To validate the hypothesis that PA66 interacts with Au NPs, ATR-FTIR spectrometry measurements were conducted on PA66 and PA66/Au NP hybrid powders (Figure 6). The characteristic peaks of PA66 and PA66 coated with Au NPs have been identified and listed in Table 1. Characteristic vibration frequency peaks of PA66 are found at 3,298 (N-H stretching), 2,933 (CH_2 _stretching), 1,631 (C = O stretching, amide I), 1,536 (N-H bending vibration) and 686 cm^-1 ^(N-H bending vibration) [23]. The presence of Au NPs at the surface of the PA66 spherulite did not change the vibration frequency of the carbonyl group of PA66 but slightly shifts the vibration frequency of the amine group (Table 1). This variation could indicate that Au NPs are interacting with the amine group of PA66 via physical bonds.

**Table 1:** Characteristic vibrations of PA66 and PA66 coated with Au NPs

Characteristic groups	PA66 (cm^-1^)	PA66 coated with Au NPs (cm^-1^)
N-H Stretching vibration	3,298	3,396
N-H Bending vibration	686	684
C = O Stretching vibration	1,631	1,631
N-H Bending vibration	1,536	1,533
-CH_2_- Stretching vibration	2,933	2,933

PA66, polyamide 66; Au NPs, gold nanoparticles.

**Figure 7:**
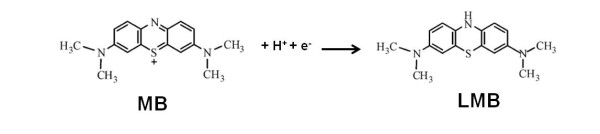
**Reduction reaction of MB to LMB**.

**Figure 8:**
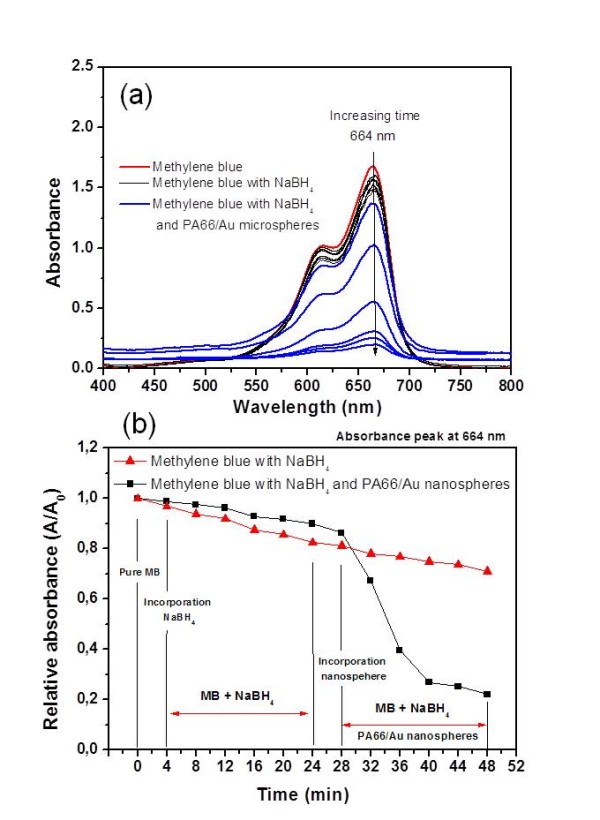
**Reduction of MB with sodium borohydride and PA66/Au NP hydrid materials**. (a) UV-Vis absorbance spectra of MB reduced by NaBH_4 _and catalysed with PA66/Au NP microspheres in the wavelength range of 400 to 800 nm. (b) Plots of the relative absorbance of MB absorption peak at 664 nm as a function of the reaction time.

PA66 spherulites coated with Au NPs as a catalyst were demonstrated by investigating the reduction of MB to leuco MB (LMB) (Figure 7) as a function of time by UV-Vis spectrometry in the wavelength range between 400 and 800 nm (Figure 8) at room temperature. To understand the effect of the hybrid material on the reduction rate of MB, further investigations need to be conducted regarding the amount of the metallised PA66 microspheres and the temperature.

In chemistry, this substance is recognised as a redox indicator since it can easily change its colour in a specific environment [4,24]. Indeed, MB, initially blue in an oxidizing environment, undergoes a definite colour change by becoming colourless in the presence of a reducing agent such as sodium borohydride [25]. The MB reduction reaction, leading to the formation of LMB, is described in Figure 7[25].

In aqueous medium, MB exhibits a main absorption peak at 664 nm with a shoulder at 614 nm as shown in Figure 8. It has been reported that the main absorption peak at 664 nm corresponds to the n-π* transition of MB [26,27]. The reduction of MB as a function of time has been investigated in the presence of sodium borohydride. Relative absorbance of the peak at 664 nm is plotted as a function of time to evaluate the MB reduction reaction rate (Figure 8b). Incorporation of the reducing agent into the MB solution decreases slightly the absorbance intensity of the peak at 664 nm with the time (Figure 8b, see Additional file 2). This decreasing trend indicates that MB starts to reduce in the presence of NaBH_4_; however, the reaction is slow. After 20 min, the PA66/Au NP hybrid system was incorporated into the MB/NaBH_4 _solution. Interestingly, a strong decrease of the UV-Vis absorbance intensity of MB is observed in the presence of the hybrid material (Figure 8a, b). Additionally, the plot of the relative absorbance of the peak at 664 nm reveals that the complete reduction of MB to LMB is accomplished in less than 20 min in the presence of the hybrid materials since the curve tends to stabilise at the end (Figure 8b). This result confirms that PA66/Au NP hybrid microspheres act as an effective catalyst in the reduction of MB.

**Figure 9:**
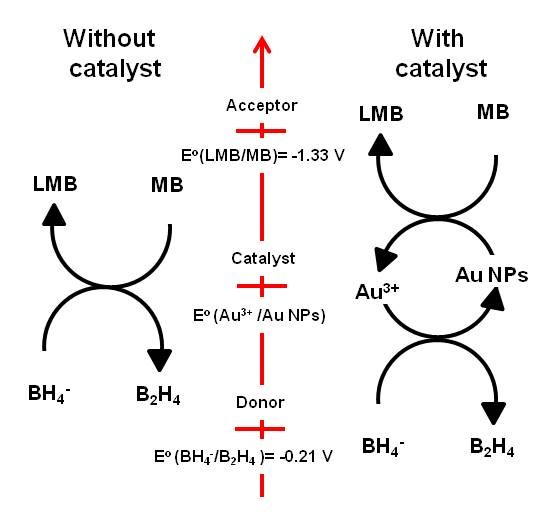
**Electron-transfer process mechanism**. The scheme is inspired from the model proposed by Mallick et al. [17] between MB and BH_4_^- ^with or without the presence of a catalyst (Au NPs).

The catalytic ability of the coated PA66 spherulites depends on the size of Au NPs produced. Indeed, gold in bulk state is chemically inert since the redox potential of this noble metal is positive [15]. It has been reported by Haruta et al. that gold is becoming catalytically active for many chemicals at a nanoscale level (diameter below 10 nm) due to the reduction of its redox potential to a negative value [19,28,29]. Thus, to act as an effective catalyst, the redox potential of Au NPs needs to be found between the redox potential of the donor and the acceptor system [17,30]. In this case, noble metal nanoparticles are considered as an electron relay in the redox reaction to transfer the electron from the donor (B_2_H_4_/BH_4_^-^) to the acceptor system (LMB/MB) since Au NPs act as both donor and acceptor of electrons [17] (Figure 9). Experimental results demonstrate that PA66 metallised with Au NPs accelerates the reduction of MB because Au NPs act as an electron relay in the MB reduction reaction. Based on this observation, it is possible to deduce that the redox potential of the Au NPs produced in this investigation is located between the redox potential of MB (E°(MB/LMB) = -1.33 V) and that of sodium borohydride (E°(B_2_H_4_/BH_4_^-^) = -0.21 V) [30] (Figure 9).

## Conclusions

To summarise, a simple concept is applied to coat PA66 spherulites with Au NPs using a wet chemical method. The acidification of the solution due to the gold chloride dissociation results in the protonation of the amine group at the edge of the PA66 spherulites which favour the coordination with gold complex charged negatively. After reduction of the gold precursor, Au NPs covered the PA66 microsphere surfaces due to the physical interaction formed between both materials. PA66/Au NP hybrid material shows interesting catalytic activities. It has been found that Au NPs coated onto the PA66 spherulite act as an electron relay in the MB reduction since the redox potential of Au NPs produced is higher than the donor potential (E°(B_2_H_4_/BH_4_^-^)), but lower than the acceptor potential (E°(LMB/MB)). This approach could be applied to fabricate a variety of hybrid microspheres based on PA66 spherulites and different types of metallic nanoparticles for a wide range of catalytic and chemical applications.

## Competing interests

The authors declare that they have no competing interests.

## Authors' contributions

NC carried out the design and the characterisation of the PA66-Au NP microspheres, performed the statistical analysis and drafted the manuscript. AF, NG and CF read and contributed in the improvement of the manuscript. All authors read and approved the final manuscript.

## Supplementary Material

Additional file 1 **Investigation of the PA66 spherulite diameter measured via DLS**. Amplitude of the scattered intensity versus the hydrodynamic radius of PA66 in the water medium. Click here for file

Additional file 2 **Reduction of MB with sodium borohydride**. UV-Vis absorbance spectra of MB reduced in the wavelength range of 400 to 800 nm. Click here for file
