# Supplementary material for: Polyamide 66 microspheres metallised with in situ synthesised gold nanoparticles for a catalytic application
Source: Nanoscale Res Lett. 2012 Mar 8;7(1):182. doi: 10.1186/1556-276X-7-182 (PMC3323437; doi:10.1186/1556-276X-7-182)
Supplement: Additional file 1 — Investigation of the PA66 spherulite diameter measured via DLS. Amplitude of the scattered intensity versus the hydrodynamic radius of PA66 in the water medium. [file 1556-276X-7-182-S1.DOC]

##### Additional file 1

Polyamide 66 Microspheres Metallised with *In-Situ* Synthesised Gold nanoparticles For Catalytic Application

Nicolas Cheval, Nabil Gindy, Clifford Flowkes, Amir Fahmi *

Department of Materials, Mechanics and Structures, Faculty of Engineering, University of Nottingham, NG7 2RD, UK

### Results and discussion

Dynamic light scattering measurement was performed on PA66 powder to determine the spherulite size. PA66 powder was dispersed in distilled water using ultrasonic bath for 10 min. The measurement was carried out at 25^o^C.

Additional file 1 shows the DLS plot of PA66. The major spherulites radius has been determined at 89.7 nm.

**Additional file 1** **- Investigation of the PA66 spherulite diameter measured via DLS.** Amplitude of the scattered intensity versus the hydrodynamic radius of PA66 in the water medium
