# Supplementary material for: Polyamide 66 microspheres metallised with in situ synthesised gold nanoparticles for a catalytic application
Source: Nanoscale Res Lett. 2012 Mar 8;7(1):182. doi: 10.1186/1556-276X-7-182 (PMC3323437; doi:10.1186/1556-276X-7-182)
Supplement: Additional file 2 — Reduction of MB with sodium borohydride. UV-Vis absorbance spectra of MB reduced in the wavelength range of 400 to 800 nm. [file 1556-276X-7-182-S2.DOC]

##### Additional file 2

Polyamide 66 Microspheres Metallised with *In-Situ* Synthesised Gold nanoparticles For Catalytic Application

Nicolas Cheval, Nabil Gindy, Clifford Flowkes, Amir Fahmi *

Department of Materials, Mechanics and Structures, Faculty of Engineering, University of Nottingham, NG7 2RD, UK

### Results and discussion

Additional file 2 shows the UV-Vis spectrum of MB in the presence of sodium borohydride in the wavelength range between 400 nm and 800 nm. The experiment has been conducted during 52 min to investigate the influence of the time on the reduction of MB in the presence of sodium borohydride.

1. **Additional file 2- Reduction of MB with sodium borohydride.** UV-Vis absorbance spectra of MB reduced in the wavelength range of 400 to 800 nm

Incorporation of the reducing agent into MB solution decreases slightly the absorbance intensity of the peak at 664 nm with the time (Additional file 2). This decreasing trend indicates that MB starts to reduce in the presence of NaBH_4_, however, the reaction is slow.
